# The Role of the Right Dorsolateral Prefrontal Cortex in Phasic Alertness: Evidence from a Contingent Negative Variation and Repetitive Transcranial Magnetic Stimulation Study

**DOI:** 10.1155/2015/410785

**Published:** 2015-05-24

**Authors:** Daniela Mannarelli, Caterina Pauletti, Antonello Grippo, Aldo Amantini, Vito Augugliaro, Antonio Currà, Paolo Missori, Nicoletta Locuratolo, Maria C. De Lucia, Steno Rinalduzzi, Francesco Fattapposta

**Affiliations:** ^1^Department of Neurology and Psychiatry, Sapienza University of Rome, Viale dell'Università 30, 00185 Rome, Italy; ^2^Department of Neuroscience, AOU Careggi, Largo Brambilla 3, 50134 Florence, Italy; ^3^IRCCS Fondazione Don Carlo Gnocchi, Via di Scandicci 265, 50143 Florence, Italy; ^4^Department of Medical-Surgical Sciences and Biotechnologies, A. Fiorini Hospital, Sapienza University of Rome, Polo Pontino, Via Firenze, 04019 Terracina, Italy; ^5^Department of Neurology and Psychiatry, Neurosurgery, Sapienza University of Rome, Viale del Policlinico, 00185 Rome, Italy; ^6^Neurology and Neurophysiopathology Unit, Sandro Pertini Hospital, Via Monti Tiburtini 385, 00157 Rome, Italy

## Abstract

Phasic alertness represents the ability to increase response readiness to a target following an external warning stimulus. Specific networks in the frontal and parietal regions appear to be involved in the alert state. In this study, we examined the role of the right dorsolateral prefrontal cortex (DLPFC) during the attentional processing of a stimulus using a cued double-choice reaction time task. The evaluation of these processes was conducted by means of Event-Related Potentials (ERPs), in particular by using the Contingent Negative Variation (CNV), and repetitive 1-Hz Transcranial Magnetic Stimulation (rTMS). Transient virtual inhibition of the right DLPFC induced by real 1-Hz rTMS stimulation led to a significant decrease in total CNV and W1-CNV areas if compared with the basal and post-sham rTMS conditions. Reaction times (RTs) did not decrease after inhibitory rTMS, but they did improve after sham stimulation. These results suggest that the right DLPFC plays a crucial role in the genesis and maintenance of the alerting state and learning processes.

## 1. Introduction

Alertness provides the capacity to increase vigilance to an impending stimulus and to achieve and maintain an alerting state [1]. Two types of alertness have been described: the tonic type and the phasic type. While tonic or intrinsic alertness is defined as wakefulness and arousal, phasic alertness represents the ability to increase response readiness to a target following an external warning stimulus [2]. Phasic activity facilitates behavioural responses engaged by task-related decision processes.

A substantial body of research has investigated the brain areas involved in alertness, particularly the phasic one [1, 2]. Neuroimaging studies have revealed that a specific network in the frontal and parietal regions achieves the alert state [1, 3, 4]. The two frontal regions that appear to be involved in alertness are the dorsolateral prefrontal cortex (DLPFC) and the anterior cingulate cortex (ACC), and the phasic and intrinsic alertness are related to the norepinephrine (NE) system, through the projections from the locus coeruleus (LC) [5].

The DLPFC is involved in detecting salient events and preparing or inhibiting motor responses, moving the focus of attention, and engaging attention at the new target [1, 6]. Monitoring the occurrence of stimuli giving rise to a decrease in reaction time (RT) with increasing ISI (foreperiod effect) also depends on the integrity of the DLPFC [7–9]. Nonetheless, recent evidence suggests that the right DLPFC may act in a more executive fashion: it sustains attention and maintains a state of alertness induced by a warning cue that precedes a target by a short interval, possibly in conjunction with the ACC or other midline frontal structures [10, 11].

One valid approach to the study of alerting is the use of psychophysiological techniques, such as Event-Related Potentials (ERPs). ERPs include the Contingent Negative Variation (CNV), which was first described by Walter et al. [12]. The CNV is a slow negative shift in brain electrical potential that occurs between paired stimuli, the first of which is a warning or preparatory stimulus (S1) and the second an imperative stimulus (S2) that requires the subject to perform a response.

The CNV is an electrophysiological signature of a task-specific preparatory state that facilitates the stimulus perception and the required responses. It reflects the activation of multiple areas, mainly involved in the actual processing of S2, which compose a specific sensorimotor neural set attentionally controlled by frontoparietal networks [6, 13]. This “expectancy wave” is associated with selective behavioral functions, such as attention, preparation, estimation, and voluntary motor control [14–16]. The CNV reflects at least two distinct associative functions: attentive orientation to the warning cue and anticipatory attention during an executive control [15, 17]. CNV can be obtained at short interval and when the interval is long (>1.5 sec) it is possible to distinguish two psychophysiological components: the early CNV (initial epoch of 500–700 ms after the warning stimulus S1) and the late CNV (200 ms preceding S2) [18, 19]. Moreover, when the imperative stimulus (S2) involves a multiple choice, it is possible to detect post-S2 components that reflect the activation of selective and executive attentional channels and inhibitory control [20].

The aim of this study was to investigate, from a psychophysiological point of view, the role of the right DLPFC during attentional processing of the stimulus using a CNV paradigm in which the S2 is represented by a double-choice reaction time task. For this purpose, we delivered inhibitory rTMS stimulation to the right DLPFC in a sample of healthy subjects.

Transcranial Magnetic Stimulation (TMS) can be used to map and study perceptual, motor, and cognitive functions in the human brain [21, 22]. TMS uses a magnetic field for indirect electrical stimulation of the brain [23]. It is a noninvasive technique that can be used to study the function of specific cortical brain areas. Moreover, TMS has been shown to induce long-lasting changes in cortical excitability [24]. In particular, low-frequency rTMS (1 Hz) is known to reduce cortical excitability in targeted brain areas for several minutes after the end of stimulation [25].

We hypothesize that a virtual dysfunction in the right DLPFC interferes with the CNV phenomenon given the role played by this area in phasic alertness.

## 2. Subjects and Methods

### 2.1. Subjects

Twelve right-handed, healthy subjects (10 males, 2 females; mean age 24.4 ± 3.2 years; range 20–30 years) were enrolled from among the staff working in the Careggi Hospital of Florence to participate in a double-blind, sham-controlled crossover study. None of the subjects had a history of neurological or psychiatric disease or of head injury, and none reported consuming excessive amounts of alcohol or were taking any medication that affects the central nervous system. Written informed consent was obtained from all participants prior to the experiment and the study was approved by the local medical ethics committee.

### 2.2. Procedure

The experiment consisted of a sequence of three CNV recordings, lasting about 25 minutes, performed in the same day at three consecutive time-points (*T*0,* T*1, and* T*2) two of which followed a TMS session that lasted 30 minutes.

First, the CNV task was recorded in basal condition. Then, according to a randomized order (https://www.random.org/), either real 1 Hz rTMS or sham 1 Hz rTMS was administered. Right after the TMS sessions, the CNV task was performed again so that for each subject a basal CNV recording, a post-real TMS CNV recording and a post-sham TMS CNV recording were obtained.

Prior to the basal recording session, the BDI and STAI Y1 and Y2 tests were administered to all the participants in order to rule out anxious and depressed subjects.

The entire experimental procedure lasted about 2.5 hours.

### 2.3. rTMS Procedure

Magnetic stimulation was applied over the right DLPFC with the surface of the coil parallel to the scalp for the real stimulation and tilted at a 90° angle with only the margin of the coil in contact with the scalp for the sham condition (given the orthogonal position of the coil relative to the scalp plane, the magnetic field induced on the scalp was minimal or nil) [26]. For rTMS, we used a standard figure-of-eight coil with an outer half-radius of 75 mm (MCFB65 Butterfly Coil), connected to a MagPro X100 stimulator (Medtronic, Denmark). The biphasic pulse waveform induced a current within the brain that flowed in a posterior to anterior direction [27]. The target of the cortical stimulation was set on cranial landmarks, according to the theoretical distance between the cortical region being targeted and a reference scalp point determined by TMS (“function-guided” procedure) [28]. The hand right cortical motor area was used as a reference point. To determine the hand motor hotspot, we identified the scalp site in which single-pulse TMS produced motor evoked potentials (MEPs) of maximal amplitude in the contralateral first dorsal interosseous (FDI) [29]. The right DLPFC was thus located 5 cm anterior to the hand motor hotspot [30, 31]. Once the position for TMS had been determined, it was marked on an elastic cap worn by the patients during each rTMS session. The coil was fixed to an adjustable arm and the landmarks on the cap were repeatedly inspected during the rTMS session to ensure accurate positioning of the coil throughout the experiments on the same day. The procedures used to determine the position of the M1 area and the motor threshold were repeated before each rTMS session.

Each rTMS session consisted of 1800 pulses at a frequency of 1 Hz, at 80% of the individual Resting Motor Threshold (RMT), which was determined for each subject according to standardized criteria [29]. On the basis of data that have emerged from behavioral TMS studies regarding the after-effects of a 1 Hz protocol, the duration of stimulation was designed to ensure that the inhibition effect lasted throughout the subsequent CNV task [21].

Participants were told that they would receive rTMS on two occasions and that it would be administered at slightly different intensity levels. Neither the subjects nor the ERP investigators, with the exception of the investigator applying the rTMS, were aware of whether real or sham stimulation was being performed. The subjective sensations of coil-scalp contact and discharge noise during sham stimulation were similar to those of the real stimulation. Since the skin sensation induced by the TMS pulse is different between real and sham stimulation, a postexperiment interview was conducted and confirmed that the subjects did not perceive any significant physical differences between the two stimulation conditions. The setting falls well within the safety guidelines [21].

### 2.4. EEG Recording

Participants were seated in an anatomic chair in a partially soundproof, faradized, and light-attenuated room. The electrophysiological signals were recorded by means of a 21-channel carbon cap with active electrodes at the F3, Fz, F4, C3, Cz, C4, P3, Pz, and P4 sites, according to the International 10–20 System, referred to linked mastoids, and grounded at the forehead. The bipolar electrooculogram (EOG) was recorded from above and below the left eye. To reduce residual TMS-related artifacts, the impedance at each electrode was kept below 3 kΩ. EEG signals and EOG were filtered using a 0.01–30 Hz bandpass. A notch filter was also applied. The data were digitized with an analog/digital (A/D) converter at a sampling rate of 1024 Hz and stored on a hard disk. A Mizar Sirius EEG-EP multifunctional system was used.

### 2.5. Event-Related Potentials: CNV

The CNV task consisted of a sequence of two paired stimuli: a first stimulus (warning: S1) that informed the subject to expect a second stimulus (imperative: S2). The warning and imperative stimuli consisted, respectively, of a flash of 100 *μ*s-1.5 J (S1) (delivered by a strobe lamp at a distance of 30 cm from the subject) and, 1750 ms later, of a sound (S2) at an intensity of 80 dB SPL, lasting 200 ms, which was randomly presented at 1000 Hz (standard-S2; *p* = 0.8) or 2000 Hz (target-S2; *p* = 0.2). The subject was instructed to push a button, held in the right hand, as quickly as possible upon hearing the 2000 Hz sound, as requested by the double-choice reaction time task for paired stimuli. The intertrial interval varied randomly between 6 and 12 s. A total of 100 trials were acquired. The task lasted about 25 minutes.

### 2.6. ERP Analysis

Trials containing eye movements (including blinks) that exceeded ±100 *μ*V in the eye channels were automatically rejected online, according to clinical guidelines [32]. Trials containing drift with deflections exceeding ±100 *μ*V in any channel were also excluded. A further selection was performed in the offline analysis to reject other kinds of artifacts not detected by the automatic rejection procedure (eye movements, erratic general movements of the patient, saturating DC shift of the trace, etc.).

The ERPs were evaluated for each subject in each of the three experimental conditions: basal, post-sham rTMS, and post-real rTMS. The analysis epoch for each CNV was 5 s with a 500 ms prestimulus baseline before S1. The CNV amplitude was measured as the total area (negative shift between S1 and S2) and as two temporal windows of interest: the early orienting window: W1 (between 500 and 700 ms following S1), and the late window: W2 (200 ms preceding S2) compared with the prestimulus baseline [18, 19]. Additional post-S2 components were identified separately for target and standard stimuli. The N1 component was defined as the most negative peak between 75 and 140 ms after the S2 stimuli, while the P3-like wave was defined as the largest positive deflection following the P200 wave that occurred at least 250 ms after the S2 stimuli [20]. Baseline-to-peak measurements for post-S2 components were computed in relation to the baseline commencing 100 ms before the S2-stimulus onset (in order to avoid CNV effects). The mean reaction times of correct responses and the accuracy of responses were calculated for each recording session (correct responses ranged between 180 and 1000 ms).

### 2.7. Statistical Analysis

Data are expressed as the mean (±1 standard deviation) for continuous variables and as proportions for categorical variables. The Kolmogorov-Smirnov test was applied to assess the normal distribution of the data and to ensure that the assumption of normality was not violated for any of the data.

In order to avoid the order effect for EEG data, given the consecutive within-design at one day, CNV parameters (total area, W1, and W2) were analysed separately by means of a factorial repeated measures ANOVA, with the experimental “condition” (baseline, real rTMS, sham rTMS) and the “electrode” (F3, Fz, F4, C3, Cz, C4, P3, Pz, and P4) as the within-subject factors and the order in which subjects received rTMS (“group” factor: group 1: basal (*T*0), sham (*T*1), and real (*T*2); group 2: basal (*T*0), real (*T*1), and sham (*T*2)) as the between-subject factor.

Post-S2 components (N1- and P3-like amplitudes and latencies) were analysed separately by means of a factorial repeated measures ANOVA, with the “condition” (baseline, real rTMS, and sham rTMS), the “electrode” (F3, Fz, F4, C3, Cz, C4, P3, Pz, and P4), and the “stimulus” (target, standard) as the within-subject factors, and the “group” factor as the between-subject factor.

A post hoc correction according to Bonferroni was then applied. Degrees of freedom were adjusted, when necessary, using the Greenhouse-Geisser epsilon coefficient for possible violations of the sphericity assumption and corrected *p* values are reported; the original degrees of freedom are reported together with their correction factor epsilon.

Owing to the effect exerted by task repetitions on behavioural performance (RT), the RT mean values were compared by using a factorial repeated measures ANOVA, with the “condition” (baseline, real rTMS, and sham rTMS) as the within-subject factor and the “group” factor as the between-subject factor. A post hoc correction according to Bonferroni was applied when necessary.

The Pearson correlation test was performed to assess any correlations between CNV areas (expressed in absolute values) and RT. A *p* < 0.05 was considered statistically significant. All the analyses were performed using the SPSS statistical package (Version 20.0).

## 3. Results

### 3.1. Psychophysiological Evaluation

**Figure 1:**
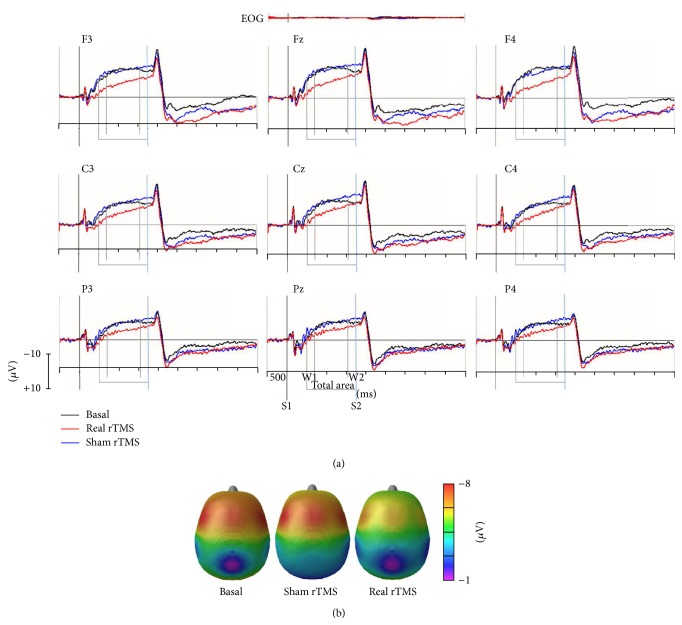
(a) Grand averaged CNV waveforms, with W1, W2, and total areas highlighted and superimposed at basal condition (black lines), post-real rTMS (red lines), and post-sham rTMS (blue lines) collapsed for condition for all subjects. S1: warning stimulus (flash). S2: imperative stimulus (tone; standard: 1000 Hz, target: 2000 Hz). (b) Scalp potential maps at 600 ms (mean value of W1-CNV) for basal condition, post-sham rTMS and post-real rTMS.

**Figure 2:**
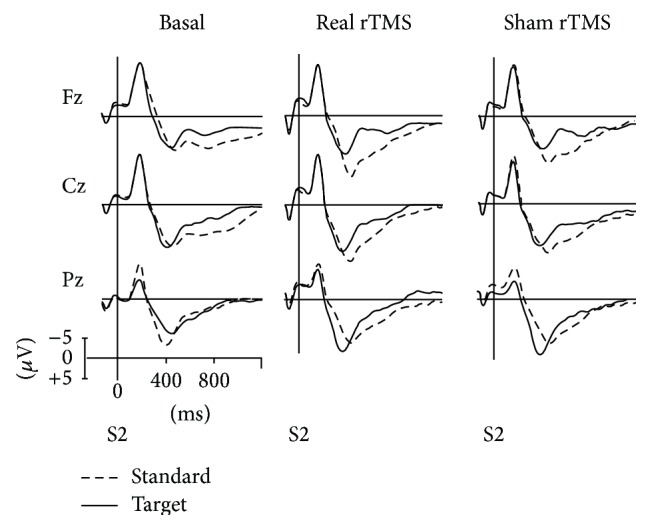
ERPs traces in midline scalp locations for target and standard stimulus at basal condition, post-real rTMS, and post-sham rTMS collapsed for condition for all subjects. The analysis epoch was 1.3 s with a 100 ms prestimulus baseline before stimulus. ERPs were pass-filtered (1–16 Hz).

CNV was elicited in 100% of the subjects. In one subject, the electrophysiological data set for the post-sham CNV could not be evaluated owing to the high artifact rate. Figures 1 and 2 show the Grand Average potentials obtained for all subjects collapsed for the three experimental conditions.

**Figure 3:**
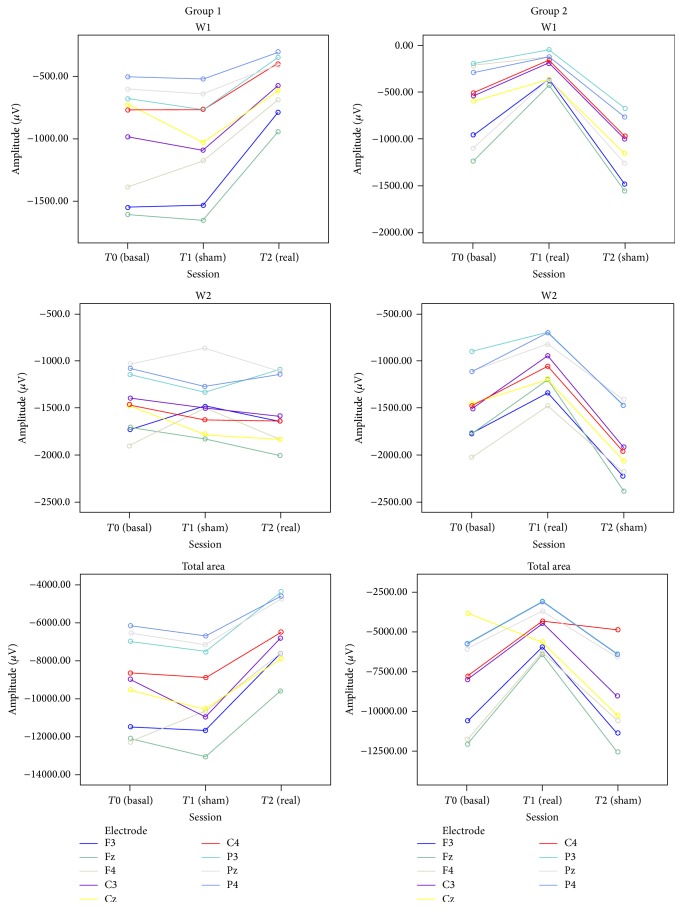
Mean amplitudes of early CNV (W1), late CNV (W2), and total CNV area across the active electrodes in basal condition, post-real rTMS, and post-sham rTMS for group 1 and group 2.

CNV Parameters: no significant difference emerged between groups (i.e., the order by which the rTMS was delivered) for both W1 (*F*_(1,7)_ = 0.37, *p* = 0.56), W2 (*F*_(1,7)_ = 0.0003, *p* = 0.98), and total area (*F*_(1,7)_ = 0.73, *p* = 0.42). A significant main effect of the “condition” factor was observed for total area and W1 (*F*_(2,14)_ = 11.21, *p* = 0.001; *F*_(2,14)_ = 11.64, *p* = 0.001, resp.). After Bonferroni correction for multiple comparisons, a reduction in amplitudes emerged only after real rTMS both for total area (basal versus real *p* = 0.01; basal versus sham *p* = 1.0; sham versus real *p* = 0.01; means (*μ*V) – basal: −8514.8; sham: −9128.4; real: −5606.8) and for W1 (basal versus real *p* = 0.05; basal versus sham *p* = 0.30; sham versus real *p* = 0.009; means (*μ*V) – basal: −783.6; sham: −1045.2; real: −378.8). By contrast, ANOVA did not yield a significant main effect of the “condition” factor for W2 (*F*_(2,14)_ = 2.37, *p* = 0.13). A significant effect of the “electrode” factor was observed for each parameter (total area: *F*_(8,56)_ = 10.21, *p* = 0.002, *ε* = 0.24; W1: *F*_(8,56)_ = 8.22, *p* = 0.015, *ε* = 0.95; W2: *F*_(8,56)_ = 20.71, *p* < 0.001). After Bonferroni correction, a significant difference emerged between frontal and parietal electrodes, with lower amplitudes being observed in the latter, for total area, W1, and W2 (Figure 3). A significant effect of the “condition” × “electrode” interaction emerged for total area (*F*_(16,112)_ = 1.80, *p* = 0.05); after Bonferroni correction, a significant difference emerged for each electrode between the real rTMS condition and basal condition as well as between the real rTMS condition and sham condition, while no difference emerged between the basal and sham conditions. The same findings emerged for W1 (*F*_(16,112)_ = 3.88, *p* = 0.016; *ε* = 0.240), though not for W2 (*F*_(16,112)_ = 1.14, *p* = 0.33).

Post-S2 parameters: no significant difference emerged between groups for post-S2 parameters (N1 amplitude: *F*_(1,6)_ = 0.07, *p* = 0.79; N1 latency: *F*_(1,6)_ = 0.12, *p* = 0.74; P3-like amplitude: *F*_(1,5)_ = 0.87, *p* = 0.39; P3-like latency: *F*_(1,5)_ = 0.67, *p* = 0.45). ANOVA did not reveal a main effect of the “condition” factor for either the N1 amplitude (*F*_(2,12)_ = 0.23, *p* = 0.80) or the P3-like latency (*F*_(2,10)_ = 0.53, *p* = 0.60) and amplitude (*F*_(2,10)_ = 0.36, *p* = 0.70). ANOVA detected a main effect of the “condition” factor only for N1 latency (*F*_(2,12)_ = 16.89, *p* < 0.001), with shorter latencies being detected in the real rTMS condition; the *p* values after Bonferroni correction were as follows: basal versus rTMS *p* = 0.001; basal versus sham *p* = 0.02; sham versus real *p* = 0.42.

There was no significant effect of the “condition” × “electrode”, of the “condition” × “stimulus,” or of the “condition” × “electrode” × “stimulus” interactions on either N1 latency (*F*_(16,96)_ = 0.48, *p* = 0.96; *F*_(2,12)_ = 6.52, *p* = 0.06; *F*_(16,96)_ = 0.72, *p* = 0.76, resp.) or amplitude (*F*_(16,96)_ = 1.47, *p* = 0.18, *ε* = 0.18, *F*_(2,12)_ = 2.98, *p* = 0.10; *F*_(16,96)_ = 0.58, *p* = 0.89, resp.) or P3-like wave latency (*F*_(16,80)_ = 0.85, *p* = 0.62; *F*_(2,10)_ = 0.37, *p* = 0.63; *F*_(16,80)_ = 0.56, *p* = 0.90, resp.) and amplitude (*F*_(16,80)_ = 0.59, *p* = 0.88; *F*_(2,10)_ = 0.09, *p* = 0.91; *F*_(16,80)_ = 0.85, *p* = 0.62, resp.). Moreover, ANOVA did not reveal a main effect on N1 and P3 amplitudes of either the “stimulus” factor (*F*_(1,6)_ = 0.03, *p* = 0.86; *F*_(1,5)_ = 0.02, *p* = 0.89, resp.) or the “stimulus” × “electrode” interaction (*F*_(8,48)_ = 3.29, *p* = 0.08; *F*_(8,40)_ = 1.33, *p* = 0.25, resp.).

A negative correlation emerged between RT and CNV areas (total area-F3: *r* = −0.41, *p* = 0.02; W1-F3: *r* = −0.38, *p* = 0.03; W2-F3: *r* = −0.38, *p* = 0.03; total area-Fz: *r* = −0.36, *p* = 0.04; W1-Fz: *r* = −0.32, *p* = 0.07; W2-Fz: *r* = −0.35, *p* = 0.048; total area-F4: *r* = −0.24, *p* = 0.2; W1-F4: *r* = −0.24, *p* = 0.18; W2-F4: *r* = −0.23, *p* = 0.19; total area-C3: *r* = −0.49, *p* = 0.004; W1-C3: *r* = −0.29, *p* = 0.10; W2-C3: *r* = −0.42, *p* = 0.02; total area-Cz: *r* = −0.07, *p* = 0.68; W1-CZ: *r* = −0.18, *p* = 0.32; W2-Cz: *r* = −0.42, *p* = 0.02; total area-C4: *r* = −0.01, *p* = 0.95; W1-C4: *r* = −0.15, *p* = 0.42; W2-C4: *r* = −0.38, *p* = 0.03; total area-P3: *r* = −0.46, *p* = 0.08; W1-P3: *r* = −0.32, *p* = 0.07; W2-P3: *r* = −0.41, *p* = 0.01; total area-Pz: *r* = −0.44, *p* = 0.01; W1-PZ: *r* = −0.15, *p* = 0.39; W2-Pz: *r* = −0.317, *p* = 0.08; total area-P4: *r* = −0.41, *p* = 0.02; W1-P4: *r* = −0.02, *p* = 0.91; W2-P4: *r* = −0.44, *p* = 0.01).

### 3.2. Behavioural Performance

**Figure 4:**
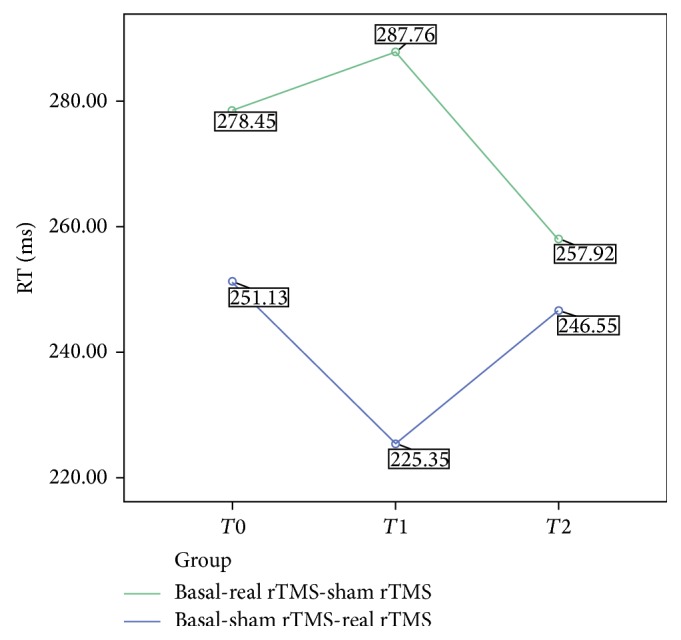
Trend of RTs for group 1 (basal, sham, and real) and group 2 (basal, real, and sham) during repetition of CNV task at* T*0,* T*1, and* T*2 times.

ANOVA revealed a main effect of the “condition” factor for RT (*p* < 0.01), with longer values after real rTMS (means RT and SD (ms)—basal: 264.8 ± 9.8; sham: 241.6 ± 7.0; rTMS: 267.5 ± 7.5); the *p* values after Bonferroni correction were as follows: basal versus real *p* = 1; basal versus sham *p* = 0.006; sham versus real *p* < 0.001. With regard to the between-subject factor, a difference bordering on significance emerged between groups (*p* = 0.056), with shorter RTs in group 1 (241.01 versus 274.08). No main effect emerged for the “condition” × “group” interaction (*F*_(2,18)_ = 0.97; *p* = 0.39) (Figure 4). Correct responses were comparable between task repetitions.

## 4. Discussion

The aim of this study was to examine whether transient inhibition of the right DLPFC interferes with the CNV phenomenon and post-S2 activities.

The following methodological issue should be taken into account before the possible implications of our results are discussed: on the basis of data that have emerged from behavioral TMS studies regarding the after-effects of a 1 Hz protocol, we chose a duration of stimulation that would ensure an inhibition effect throughout the subsequent CNV task [21]. Moreover, in the absence of neuronavigation TMS coil positioning, we defined the DLPFC cortex as lying 5 cm anterior to the region from which the most prominent motor response of the muscle FDI is recorded. This approach, which is one of the possible methods for locating a specific brain area [33], is derived from previous studies on the DLPFC [30, 34].

As hypothesized, the functional inhibition induced by low-frequency rTMS on the right DLPFC significantly reduced the overall area of the CNV waveforms in all the recording sites, particularly in the frontocentral areas. Moreover, the early CNV area was also reduced following inhibition of the right DLPFC.

These electrophysiological changes point to the suppression of the cerebral activity believed to prepare the system for a rapid response after a warning signal [35] and suggest that the right DLPFC may play a key role in the initiation and maintenance of a phasic alerting burst. The decrease in the CNV area does not appear to be related to the attenuation or habituation processes usually reported to be associated with the repetition of a CNV motor task [36, 37]. Indeed, a recent study demonstrated that the repetition of a double-choice CNV task similar to ours requires the continual recruitment of attentional resources, which prevents the occurrence of habituation phenomena [38].

The reduction in CNV we observed confirms that the right DLPFC is involved in the functioning of alerting, as previous neuroimaging studies have also shown. Critchley et al. [39] suggested that the right DLPFC is activated during preparatory and anticipatory arousal. More recently, Fan et al. [40] observed, upon studying response anticipation and preparation functions, the activation of an extensive thalamo-cortico-striatal network, including regions such as the middle and superior frontal gyrus, parietal cortex, and the prefrontal cortices (PFC), with a predominant lateralization on the right.

Our experiment also revealed that the CNV phenomenon was reduced bilaterally, which indicates that the inhibitory cortical effect was widespread even though rTMS stimulation was unilateral. This widespread reduction in CNV yields further evidence of the capacity of the right DLPFC to achieve cognitive integration during sustained attentional processing of a stimulus, which may be ascribed to the activation of bilateral neural areas. The PFC is known to consist of a set of neocortical areas that have bidirectional links with several brain cortical regions, including the sensory and motor areas [41], and a wide range of subcortical structures with widespread projections, such as the basal ganglia [42] and ACC [43, 44]. This places the PFC in an ideal anatomical position for monitoring numerous cognitive processes, including phasic alertness, and for synthesizing the wide range of information needed for complex sensorimotor acts [41].

It is noteworthy that the functional inhibition of the right DLPFC does not interfere with the late CNV area, which indicates that the electrical activity related to motor readiness is not directly modified by the virtual lesion in this area. We therefore believe that, during a motor task, the DLPFC sustains the activation of sensory and motor cortices by maintaining a state of alertness induced by a warning signal, without interfering with preprogramming and motor preparation.

Interestingly, the post-S2 components did not change significantly after inhibitory rTMS stimulation. N1 latency became even shorter over repetitions, likely reflecting a growing confidence in stimulus discrimination [45, 46]. The stability of the P3-like components leads us to speculate that the right DLPFC may not directly influence the executive discrimination processes evoked during a task such as ours, which required an imperative, discriminative, and inhibitory motor response. We believe that these functions were not modified as they are likely to have been supplied by the left hemispheric cortices, which are known to be involved in single event processing and motor inhibition [47, 48].

The relationship between the right DLPFC and motor performances also deserves consideration. It is known that the repetition of a CNV motor task like ours is associated with a reduction in RTs, which is probably due to the maintenance of high levels of attention displayed by high CNV amplitudes over sessions [38, 49]. The results related to RT in the present study seem to confirm this observation: RTs were significantly shorted after sham rTMS (which always follows at least one execution of the task) than at the basal CNV recording which indicates that repetition of the CNV task led to the motor performance being learned. This procedural learning was closely related to the maintenance of a higher attentional performance, as demonstrated by the high CNV amplitude after sham rTMS. By contrast, when the degree of attentional processing was reduced (low CNV amplitude after real rTMS on the right DLPFC), the RTs did not decrease. Even if in the current experiment the lack of unwarned trials does not allow giving a direct evidence of the role of large CNV on the RT improvement, our results suggest that the right DLPFC may interfere with the learning of a motor performance through direct interference in attentional functioning and in particular on the ability to orient and to sustain the alerting response to a warning stimulus, which is critical to the learning process.

## 5. Conclusion

Although the results of our psychophysiological study should be considered as preliminary owing to the limited sample size and the nature of the procedure adopted, they do provide noteworthy findings on the anatomofunctional correlates of sustained phasic alertness. Further studies conducted on larger samples and using more sophisticated techniques are warranted to confirm these findings. Lastly, the results of our study strongly indicate that the right DLPFC plays a critical role in the genesis and maintenance of the alerting state.

## References

[1] Posner M. I., Petersen S. E. (1990). The attention system of the human brain. *Annual Review of Neuroscience*.

[2] Petersen S. E., Posner M. I. (2012). The attention system of the human brain: 20 years after. *Annual Review of Neuroscience*.

[3] Pardo J. V., Fox P. T., Raichle M. E. (1991). Localization of a human system for sustained attention by positron emission tomography. *Nature*.

[4] Coull J. T., Nobre A. C., Frith C. D. (2001). The noradrenergic *α*2 agonist clonidine modulates behavioural and neuroanatomical correlates of human attentional orienting and alerting. *Cerebral Cortex*.

[5] Aston-Jones G., Cohen J. D. (2005). An integrative theory of locus coeruleus-norepinephrine function: adaptive gain and optimal performance. *Annual Review of Neuroscience*.

[6] Corbetta M., Shulman G. L. (2002). Control of goal-directed and stimulus-driven attention in the brain. *Nature Reviews Neuroscience*.

[7] Stuss D. T., Alexander M. P., Shallice T. (2005). Multiple frontal systems controlling response speed. *Neuropsychologia*.

[8] Vallesi A., Shallice T., Walsh V. (2007). Role of the prefrontal cortex in the foreperiod effect: TMS evidence for dual mechanisms in temporal preparation. *Cerebral Cortex*.

[9] Vallesi A., McIntosh A. R., Shallice T., Stuss D. T. (2009). When time shapes behavior: fMRI evidence of brain correlates of temporal monitoring. *Journal of Cognitive Neuroscience*.

[10] Critchley H. D., Melmed R. N., Featherstone E., Mathias C. J., Dolan R. J. (2002). Volitional control of autonomic arousal: a functional magnetic resonance study. *NeuroImage*.

[11] Critchley H. D., Mathias C. J., Josephs O. (2003). Human cingulate cortex and autonomic control: converging neuroimaging and clinical evidence. *Brain*.

[12] Walter W. G., Cooper R., Aldridge V. J., McCallum W. C., Winter A. L. (1964). Contingent negative variation: an electric sign of sensori-motor association and expectancy in the human brain. *Nature*.

[13] Gómez C. M., Flores A. (2011). A neurophysiological evaluation of a cognitive cycle in humans. *Neuroscience and Biobehavioral Reviews*.

[14] McCallum W. C., Walter W. G. (1968). The effects of attention and distraction on the contingent negative variation in normal and neurotic subjects. *Electroencephalography and Clinical Neurophysiology*.

[15] Tecce J. J. (1972). Contingent negative variation (CNV) and psychological processes in man. *Psychological Bulletin*.

[16] Birbaumer N., Elbert T., Canavan A. G. M., Rockstroh B. (1990). Slow potentials of the cerebral cortex and behavior. *Physiological Reviews*.

[17] Cui R. Q., Egkher A., Huter D., Lang W., Lindinger G., Deecke L. (2000). High resolution spatiotemporal analysis of the contingent negative variation in simple or complex motor tasks and a non-motor task. *Clinical Neurophysiology*.

[18] Gaillard A. W. K. (1976). Effects of warning-signal modality on the contingent negative variation (CNV). *Biological Psychology*.

[19] Travis F., Tecce J. J. (1998). Effects of distracting stimuli on CNV amplitude and reaction time. *International Journal of Psychophysiology*.

[20] Golob E. J., Pratt H., Starr A. (2002). Preparatory slow potentials and event-related potentials in an auditory cued attention task. *Clinical Neurophysiology*.

[21] Rossi S., Hallett M., Rossini P. M. (2009). Safety, ethical considerations, and application guidelines for the use of transcranial magnetic stimulation in clinical practice and research. *Clinical Neurophysiology*.

[22] Miniussi C., Thut G. (2010). Combining TMS and EEG offers new prospects in cognitive neuroscience. *Brain Topography*.

[23] Barker A. T., Jalinous R., Freeston I. L. (1985). Non-invasive magnetic stimulation of human motor cortex. *The Lancet*.

[24] Pascual-Leone A., Valls-Solé J., Wassermann E. M., Hallett M. (1994). Responses to rapid-rate transcranial magnetic stimulation of the human motor cortex. *Brain*.

[25] Chen R., Classen J., Gerloff C. (1997). Depression of motor cortex excitability by low-frequency transcranial magnetic stimulation. *Neurology*.

[26] Lisanby S. H., Gutman D., Luber B., Schroeder C., Sackeim H. A. (2001). Sham TMS: intracerebral measurement of the induced electrical field and the induction of motor-evoked potentials. *Biological Psychiatry*.

[27] Kammer T., Beck S., Thielscher A., Laubis-Herrmann U., Topka H. (2001). Motor thresholds in humans: a transcranial magnetic stimulation study comparing different pulse waveforms, current directions and stimulator types. *Clinical Neurophysiology*.

[28] Sparing R., Buelte D., Meister I. G., Pauš T., Fink G. R. (2008). Transcranial magnetic stimulation and the challenge of coil placement: a comparison of conventional and stereotaxic neuronavigational strategies. *Human Brain Mapping*.

[29] Rossini P. M., Barker A. T., Berardelli A. (1994). Non-invasive electrical and magnetic stimulation of the brain, spinal cord and roots: basic principles and procedures for routine clinical application. Report of an IFCN committee. *Electroencephalography and Clinical Neurophysiology*.

[30] Pascual-Leone A., Rubio B., Pallardó F., Catalá M. D. (1996). Rapid-rate transcranial magnetic stimulation of left dorsolateral prefrontal cortex in drug-resistant depression. *The Lancet*.

[31] George M. S., Wassermann E. M., Williams W. A. (1996). Changes in mood and hormone levels after rapid-rate transcranial magnetic stimulation (rTMS) of the prefrontal cortex. *The Journal of Neuropsychiatry & Clinical Neurosciences*.

[32] Duncan C. C., Barry R. J., Connolly J. F. (2009). Event-related potentials in clinical research: guidelines for eliciting, recording, and quantifying mismatch negativity, P300, and N400. *Clinical Neurophysiology*.

[33] Sandrini M., Umiltà C., Rusconi E. (2011). The use of transcranial magnetic stimulation in cognitive neuroscience: a new synthesis of methodological issues. *Neuroscience and Biobehavioral Reviews*.

[34] George M. S., Wassermann E. M., Williams W. A. (1995). Daily repetitive transcranial magnetic stimulation (rTMS) improves mood in depression. *NeuroReport*.

[35] Posner M. I. (2008). Measuring alertness. *Annals of the New York Academy of Sciences*.

[36] Timsit-Berthier M. (1984). Contingent negative variation and endogenous components of the evoked potential. *Revue d’Electroencéphalographie et de Neurophysiologie Clinique*.

[37] Rushby J. A., Barry R. J. (2007). Event-related potential correlates of phasic and tonic measures of the orienting reflex. *Biological Psychology*.

[38] Pauletti C., Mannarelli D., Grippo A. (2014). Phasic alertness in a cued double-choice reaction time task: a Contingent Negative Variation (CNV) study. *Neuroscience Letters*.

[39] Critchley H. D., Mathias C. J., Dolan R. J. (2001). Neural activity in the human brain relating to uncertainty and arousal during anticipation. *Neuron*.

[40] Fan J., Kolster R., Ghajar J. (2007). Response anticipation and response conflict: an event-related potential and functional magnetic resonance imaging study. *The Journal of Neuroscience*.

[41] Miller E. K. (2000). The prefrontal cortex and cognitive control. *Nature Reviews Neuroscience*.

[42] Pasupathy A., Miller E. K. (2005). Different time courses of learning-related activity in the prefrontal cortex and striatum. *Nature*.

[43] Frith C. D., Friston K., Liddle P. F., Frackowiak R. S. J. (1991). Willed action and the prefrontal cortex in man: a study with PET. *Proceedings of the Royal Society B: Biological Sciences*.

[44] Cohen R. A., Kaplan R. F., Zuffante P. (1999). Alteration of intention and self-initiated action associated with bilateral anterior cingulotomy. *Journal of Neuropsychiatry and Clinical Neurosciences*.

[45] Shelley A. M., Ward P. B., Michie P. T. (1991). The effect of repeated testing on ERP components during auditory selective attention. *Psychophysiology*.

[46] Benikos N., Johnstone S. J., Roodenrys S. J. (2013). Short-term training in the Go/Nogo task: behavioural and neural changes depend on task demands. *International Journal of Psychophysiology*.

[47] MacDonald A. W., Cohen J. D., Stenger V. A., Carter C. S. (2000). Dissociating the role of the dorsolateral prefrontal and anterior cingulate cortex in cognitive control. *Science*.

[48] Wood J. N., Grafman J. (2003). Human prefrontal cortex: processing and representational perspectives. *Nature Reviews Neuroscience*.

[49] Fattapposta F., Amabile G., Cordischi M. V. (1996). Long-term practice effects on a new skilled motor learning: an electrophysiological study. *Electroencephalography and Clinical Neurophysiology*.

